# Supplementary material for: A cholesterol switch controls phospholipid scrambling by G protein–coupled receptors
Source: J Biol Chem. 2024 Jan 16;300(2):105649. doi: 10.1016/j.jbc.2024.105649 (PMC10874734; doi:10.1016/j.jbc.2024.105649)
Supplement: Supporting Figures S1–S6 [file mmc1.docx]

Supplementary Information

A cholesterol switch controls phospholipid scrambling by G protein-coupled receptors

Indu Menon^1^, Taras Sych^2^, Yeeun Son^3,4^, Takefumi Morizumi^5^, Joon Lee^1^, Oliver P. Ernst^5,6^, George Khelashvili^7,8^, Erdinc Sezgin^3^, Joshua Levitz^1^, Anant K. Menon^1*^

^1^Department of Biochemistry, Weill Cornell Medical College, New York, NY 10065, USA,

^2^Science for Life Laboratory, Department of Women's and Children's Health, Karolinska Institutet, 17165 Solna, Sweden

^3^Graduate program in Biochemistry, Cell and Molecular Biology, Weill Cornell Graduate School, and ^4^Structural Biology Program, Memorial Sloan Kettering Cancer Center, New York, NY 10065, USA,

^5^Department of Biochemistry, University of Toronto, Toronto, ON, Canada M5S 1A8,

^6^Department of Molecular Genetics, University of Toronto, Toronto, ON, Canada M5S 1A8,

^7^Department of Physiology and Biophysics, and ^8^Institute of Computational Biomedicine, Weill Cornell Medical College, New York, NY 10065, USA


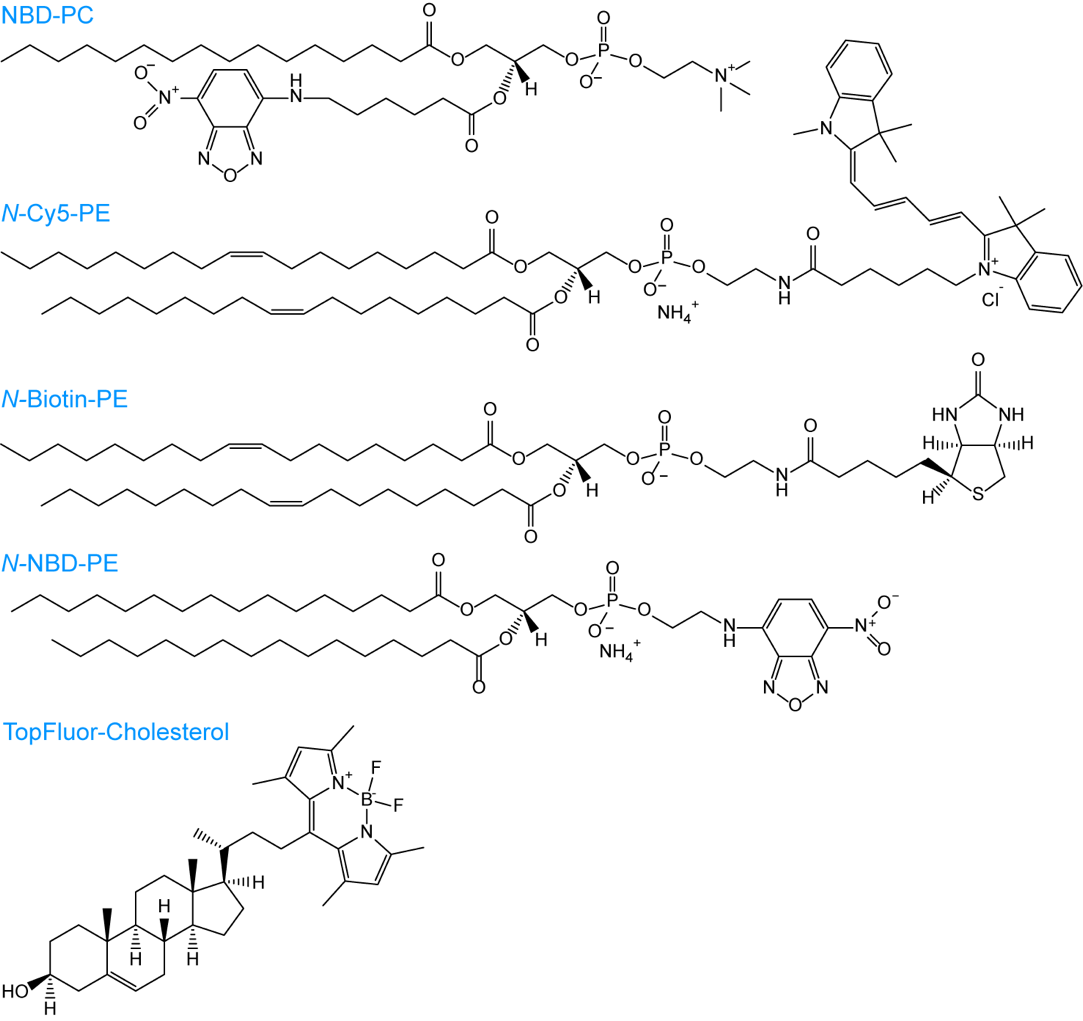


Figure S1. Fluorescent lipids used in this study.

NBD-PC is the reporter lipid used for scramblase assays. *N*-Cy5-PE, *N*-Biotin-PE and TopFluor-Cholesterol are used in the single vesicle imaging experiment shown in Fig 2. *N*-NBD-DPPE is used in Fig. S3.


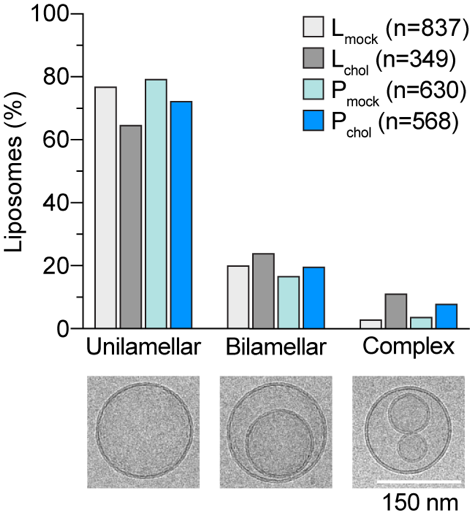


Figure S2. Morphology of mock-treated and CDC-treated vesicles determined by cryo-electron microscopy.

Protein-free liposomes (L) and opsin-containing proteoliposomes (P) were subjected to two rounds of mock-treatment to generate L_mock_ and P_mock_ samples (0% cholesterol), or CDC-treatment to generate L_chol_ and P_chol_ samples (~40% cholesterol). The samples were imaged by cryo-electron microscopy as described in Experimental Procedures. Examples of the different vesicle morphologies observed are shown in the images below the bar chart. The number of vesicles counted is indicated in the key.


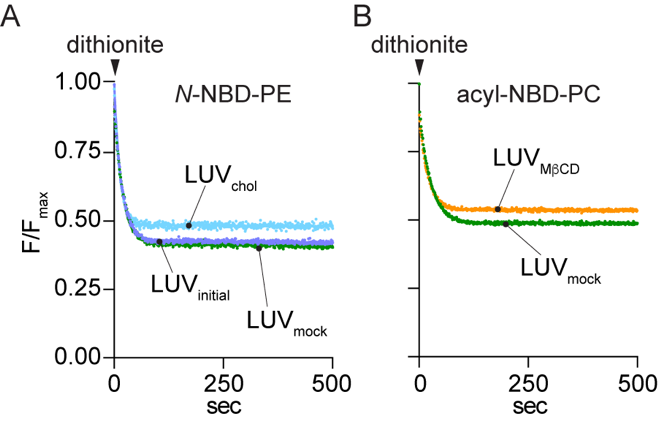


Figure S3. Controls for evaluation of the methodology for supplementing LUVs with cholesterol.

**A.** LUVs containing *N*-NBD-PE were subjected to two rounds of treatment with buffer (LUV_mock_) or CDC complex (LUV_chol_). Dithionite was added and the time course of fluorescence decay was monitored. The data were analyzed as in Fig. 3A to determine L(inside)(L_i_, defined in Fig. 3A, inset)). Exemplary traces are shown. L_i_ = 0.43, 0.43 and 0.49 for LUV_initial_, LUV_mock_, and LUV_chol_, respectively. The relative ratio of NBD fluorescence to total phospholipid was 1.0, 0.99 and 0.98 for LUV_initial_, LUV_mock_, and LUV_chol_, respectively.

**B.** LUVs containing acyl-NBD-PC were subjected to two rounds of incubation with buffer (LUV_mock_) or MβCD (LUV_MβCD_) and treated with dithionite as in Fig. 3A to determine L_i_. Exemplary traces are shown. L_i_ = 0.49 and 0.54 for LUV_mock_ and LUV_MβCD_, respectively.


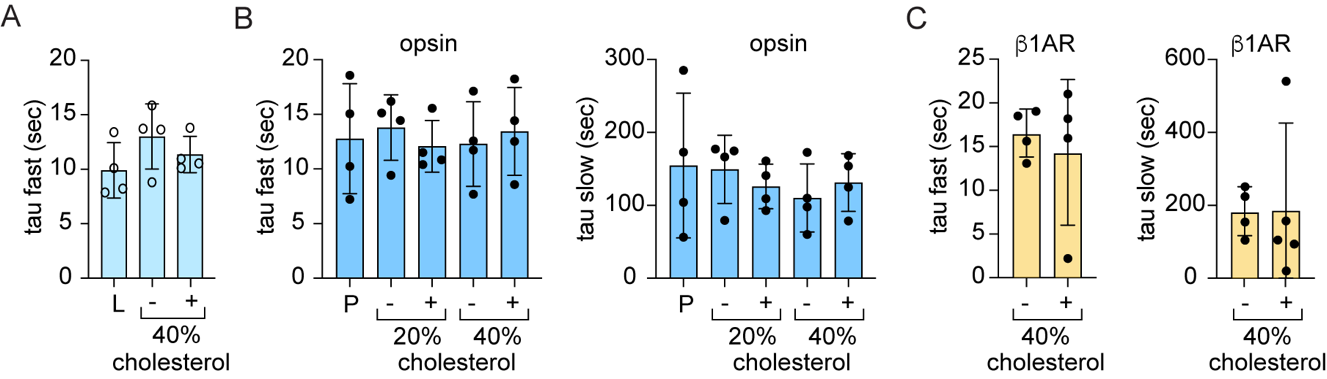


Figure S4. Kinetics of dithionite-mediated bleaching of NBD-PC in liposomes and GPCR-proteoliposomes.

Protein-free liposomes (L) and GPCR-containing proteoliposomes (P) were subjected to one or two rounds of CDC-treatment to generate samples with ~20% and ~40% cholesterol, respectively. Mock-treated vesicles were prepared in parallel. Fluorescence traces including those shown in Figs 1E, 3A, 4A and 6D,E were analyzed by fitting to exponential decay functions. The liposome data (A) were fit to a mono-exponential function with a weak linear component (<10^-4^ s^-1^). The proteoliposome data (opsin (B) and β1AR (C)) were analyzed using a double exponential function. The time constants (tau values) associated with the fits are shown. Pairwise comparisons of tau values in each data set (ordinary one-way ANOVA (panels A,B), and two-tailed, unpaired t-test (panel C)) indicated no significant differences between values.


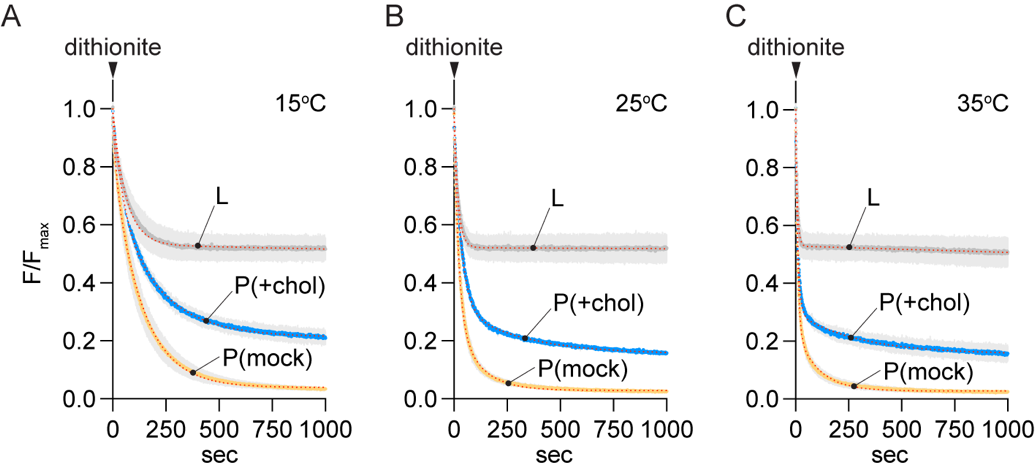


Figure S5. Cholesterol-mediated inhibition of opsin-mediated scrambling measured at different temperatures.

Fluorescence traces obtained on adding dithionite to mock-treated or CDC-treated opsin-proteoliposomes (P(mock) and P(+chol), respectively) at different temperatures as indicated. The CDC-treated sample had ~35% cholesterol. Data were obtained as described in Fig. 1D. Protein-free liposomes (L) were analyzed in parallel - for simplified presentation, as in Fig. 4, the liposome traces are shown as a single average (± S.D., error bars in light grey) of duplicate traces obtained for mock and CDC-treated liposomes. The P(mock) and P(+chol) traces are the average (± S.D., error bars in light grey) of duplicate measurements. The dotted line superimposed on the liposome data represents a mono-exponential decay model as in Fig. 3B. Dotted lines superimposed on the traces for P(mock) and P(+chol) represent double-exponential fits. The rate of dithionite-mediated bleaching of NBD fluorophores (obtained from fitting of the liposome data) increased with temperature: tau(fast) ~45, 20, 5 s for measurements at 15^o^C, 25^o^C and 35^o^C, respectively. The tau(slow) values for the proteoliposome measurements were similar at all temperatures. The ratio of the fraction of active vesicles in P(+chol) versus P(mock) vesicles obtained from the three measurements was 0.74 ± 0.05 (mean ± S.D.)), consistent with the data shown in Fig. 6C.


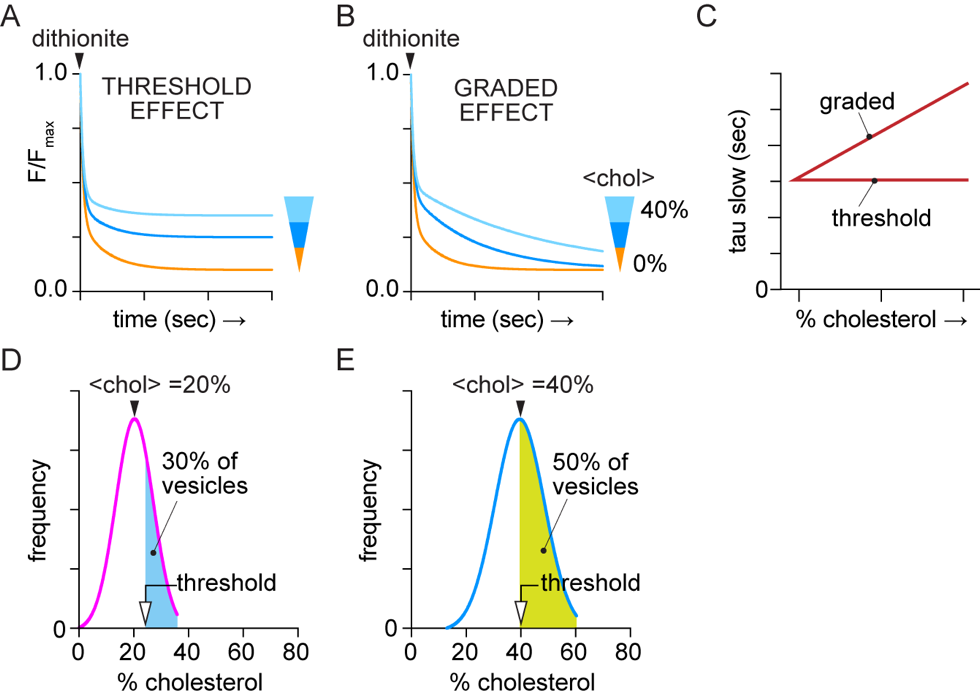


Figure S6. Predicted outcomes for threshold versus graded effect scenarios of cholesterol-mediated inhibition of GPCR scrambling.

**A.** Threshold effect scenario where tau(slow) for a fraction of the cholesterol-treated vesicles is >100-fold greater than for mock-treated vesicles, hence not readily measured on the time scale of the assay. Treated vesicles that retain scramblase activity have the same tau(slow) as mock-treated vesicles. The schematic illustration shows a set of three traces, all with tau(fast)=20, tau(slow)=200, and plateau values of 0.1 (yellow), 0.25 (dark blue), 0.35 (light blue).

**B.** A graded effect (panel A) would cause a gradual reduction in the rate of scrambling, seen as an increase in tau(slow) of a double-exponential fit (modeled here as a set of three traces, all with plateau =0.1 and tau(fast)=20, but with tau(slow)= 200 (yellow), 500 (dark blue), 1000 (light blue), in units of seconds.

**C.** Predicted outcomes for tau(slow) as a function of cholesterol concentration according to the threshold or graded effect models.

**D, E.** Models to estimate the inactivation threshold concentration of cholesterol for β1AR-mediated scrambling. The range of cholesterol concentrations in individual vesicles within the sample is presumed to follow a normal distribution. Panel D shows a schematic of a β1AR-proteoliposome sample treated once with CDC, resulting in a mean cholesterol concentration of 20%. The blue shaded area corresponds to 30% of the total area under the curve, representing the population of vesicles that is inactivated by cholesterol in this condition. Panel E shows a sample treated twice with CDC, resulting in a mean cholesterol concentration of 40%. The green shaded area corresponds to 50% of the total area under the curve, representing the population of vesicles that is inactivated by cholesterol in this condition. In both panels, the threshold concentration above which cholesterol inactivates scrambling in β1AR-proteoliposomes is indicated.
